# Shared and Distinct T‑Cell Receptor Characteristics of CD4+ and CD8+ Drug-Specific T‑Cells *In Vitro* Generated from Healthy Donors and Hypersensitive Patients

**DOI:** 10.1021/acs.chemrestox.6c00152

**Published:** 2026-08-18

**Authors:** Kanoot Jaruthamsophon, Paul J. Thomson, Jirawat Pratoomwun, Qing Zhao, Mubarak Almutairi, Antida Sangiemchoey, Araya Yeunyongviwat, Kanitpong Phabphal, Hong Liu, Furen Zhang, Yonghu Sun, Chonlaphat Sukasem, Munir Pirmohamed, Dean J. Naisbitt

**Affiliations:** † Department of Pathology, Faculty of Medicine, Prince of Songkla University, Songkhla 90110, Thailand; ‡ Centre for Drug Safety Science, Department of Pharmacology and Therapeutics, Institute of Systems, Molecular and Integrative Biology, 4591 University of Liverpool, Liverpool L69 3GE, U.K.; § Division of Pharmacogenomics and Personalized Medicine, Department of Pathology, Faculty of Medicine Ramathibodi Hospital, Mahidol University, Bangkok 10400, Thailand; ∥ Laboratory for Pharmacogenomics, Somdech Phra Debaratana Medical Center (SDMC), Ramathibodi Hospital, Bangkok 10400, Thailand; ⊥ Department of Clinical Chemistry, Faculty of Medical Technology, Huachiew Chalermprakiet University, Samut Prakan 10540, Thailand; # Shandong Provincial Hospital for Skin Diseases & Shandong Provincial Institute of Dermatology and Venereology, Shandong First Medical University & Shandong Academy of Medical Sciences, Jinan 250022, China; ¶ Department of Medical Laboratory Sciences, College of Applied Medical Sciences in Alquwayiyah, Shaqra University, Shaqra, Riyadh Province 15526, Saudi Arabia; ∇ Division of Pharmacy, Songklanagarind Hospital, Faculty of Medicine, 26686 Prince of Songkla University, Songkhla 90110, Thailand; ○ Department of Pediatrics, Faculty of Medicine, Prince of Songkla University, Songkhla 90110, Thailand; ⧫ Department of Internal Medicine, Faculty of Medicine, Prince of Songkla University, 90110 Songkhla, Thailand; †† Faculty of Pharmaceutical Sciences, Burapha University, Saensuk, Mueang, Chonburi 20131, Thailand

## Abstract

The initiation of T-cell-mediated drug hypersensitivity reaction requires the formation of a human leukocyte antigen (HLA)-peptide-drug-T-cell receptor (TCR) complex. While specific interactions between HLA molecules and drug antigens are well-defined, the characteristics of drug binding to the TCR are unclear. Herein, we sequenced the TCR mRNAs of T-cells that express drug-specific HLAs (including *HLA*-A*31:01, *HLA*-B*15:02, and *HLA*-B*13:01) and exhibit drug-specific immune responses against carbamazepine, sulfamethoxazole, and dapsone. This study included a total of 46 *in vitro*-generated monoclonal drug-specific (CD4+ and CD8+) T-cell clones. The T-cell clones were characterized for their drug-specific immune response, including HLA restriction properties, interferon-γ secretion, and cross-reactive responses. RNA isolated from these cells was used for TCR library preparation and subsequent long-read sequencing analyses. T-cell characterization studies showed different drug-responsiveness properties among the clones. TCR sequencing revealed wide heterogeneity among drug-specific T-cells. Only TCR Vβ (*TRBV*) and TCR Jβ (*TRBJ*) segments of CD4+ T-cell clones displayed noticeable shared characteristics. A YEQYF residue on CDR3β, which correlates with the preferential use of *TRBJ*2–7, was identified in 5 out of 16 CD4+ carbamazepine-specific clones. However, CD8+ drug-specific clones showed no preferential usage of TCR α/β. Sequence alignment analyses among clones with similar or different drug-responsiveness properties also showed no evidence of structure–function correlation. In summary, TCR specificity against drug antigens appears to be nonrestrictive, suggesting that the extent of possible binding and its key determinants are not rigid.

## Graphical abstract

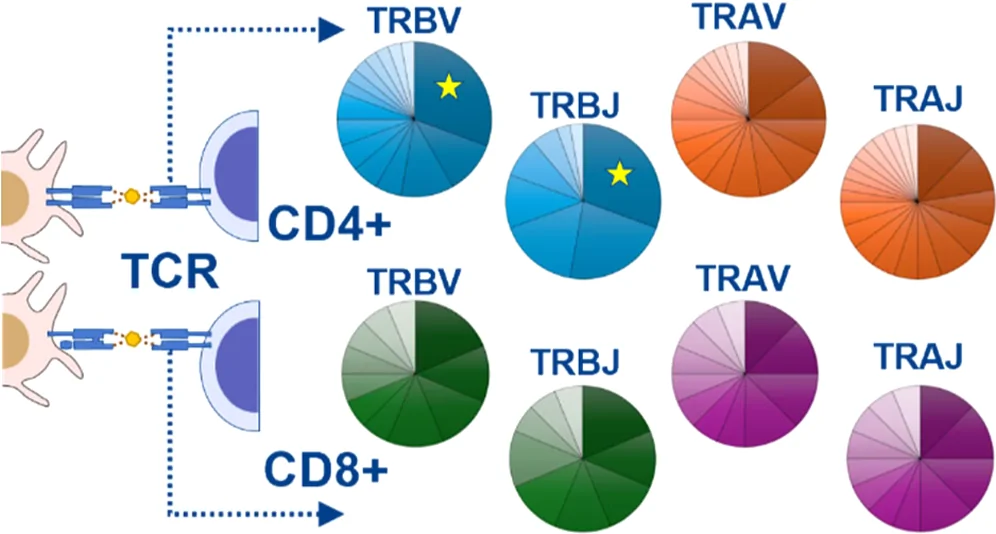

## Introduction

T cells play an effector role in the pathogenesis of delayed-type drug hypersensitivity reactions. The T-cell receptor (TCR) is a T-cell surface protein that is crucial for recognizing peptide antigens on human leukocyte antigen (HLA) molecules. TCRs exhibit immense diversity through a process called V­(D)­J recombination, with an estimated number of 10^7^ possible unique TCRs in an individual.^1^ The formation of an HLA-peptide-drug-TCR complex is a critical and complicated process in the initiation of drug hypersensitivity reactions. Certain HLA class I and II molecules have been shown to bind to drug or drug peptide complexes and present them to the TCR. For instance, in 2011, a study on carbamazepine (CBZ) hypersensitive patients carrying *HLA*-B*15:02 reported that some drug-specific TCRs are exclusive, restricted, and necessary for the pathogenesis of the reaction.^2^ However, subsequent studies revealed that TCRs recognizing the CBZ molecule are highly polymorphic.^3,4^ This raises questions regarding the extent of TCR heterogeneity underlying drug-antigen recognition and the relevance of TCRs in predicting drug hypersensitivity reactions, alongside HLA markers.

A wide variety of drug-specific T-cell populations and drug-specific TCRs have been reported in reactions caused by other medications, including allopurinol,^5,6^ dapsone (DDS),^7^ and sulfamethoxazole (SMX).^8^ A common finding in these reports is that while TCRs are highly polymorphic, they often share some common TCR Vβ features.^5,7,8^ However, less is known about how these shared TCR characteristics determine the TCR’s interaction with drugs and HLA molecules. In this study, we performed TCR sequencing analysis on *in vitro* generated monoclonal drug-specific T-cells, whose functional characteristics were extensively investigated. The objectives of the study were to (1) explore the characteristics of drug-specific TCRs and (2) determine the possible relationship between TCR properties and drug-specific T-cell responses.

## Material and Methods

### Participants and Sample Collection

Peripheral blood mononuclear cells (PBMCs) were obtained from nine participants (two healthy donors and seven drug-hypersensitive patients) for the *in vitro* generation of drug-specific T-cells (Table 1). The two healthy donors were drug-naïve individuals carrying high-risk alleles known to predispose them to CBZ-induced severe cutaneous adverse reactions (one with *HLA*-A*31:01 and one with *HLA*-B*15:02). Both healthy donor samples were retrieved from the University of Liverpool biobank cohort.

**Table 1:** Characteristics of Donors and Hypersensitive Patients^a^. High-risk HLA Markers Associated With Drug Hypersensitivity Reaction are in Bold

case no.	recruiting site	clinical information	HLA genotype	number of generated drug-specific T-cell clones
1	Liverpool, UK	CBZ-naïve healthy donor	A*01:01/A*31:01	1 CBZ CD4
			B*07:02/B*52:01	
			C*07:02/C*12:02	
			*DRB*1*15:01/*DRB*1*15:02	
2	Liverpool, UK	CBZ-naïve healthy donor	A*11:01/A*24:07	8 CBZ CD4
			B*13:02/B*15:02	
			C*04:01/C*08:01	
			*DRB*1*04:05/*DRB*1*07:01	
3	Liverpool, UK	CBZ-induced DRESS: generalized maculopapular exanthema with fever, eosinophilia, and lymphocytosis	A*11:01/A*31:01	4 CBZ CD4
			B*27:05/B*40:01	1 CBZ CD8
			C*01:02/C*03:04	
			*DRB*1*03:01/*DRB*1*04:04	
4	Liverpool, UK	CBZ-induced rash: generalized erythematous rash	A*01:01/A*24:02	10 CBZ CD4
			B*50:01/B*57:01	
			C*06:02/C*06:02	
			*DRB*1*03:01/*DRB*1*07:01	
5	Songkhla, Thailand	CBZ-induced SJS: fever, rash at arms, legs and trunk, eyes swelling, crack lips; phenytoin-induced rash: fever, erythematous rash on trunk	A*03:01/A*26:01	2 CBZ CD4
			B*07:05/B*15:02	
			C*07:02/C*08:01	
			*DRB*1*12:02/*DRB*1*15:01	
6	Bangkok, Thailand	SMX/TMP-induced DRESS: maculopapular exanthema at face and extremities, abnormal liver function tests	A*11:01/A*68:01	1 SMX CD4,
			B*13:01/B*52:01	2 SMX-NO CD4
			C*03:04/C*12:02	
			*DRB*1*15:01/*DRB*1*15:01	
7	Bangkok, Thailand	SMX/TMP-induced DRESS: confluent maculopapular exanthema on trunk and extremities, abnormal liver function tests	A*11:01/A*33:03	4 SMX-NO CD4
			B*13:01/B*58:01	
			C*03:02/C*03:04	
			*DRB*1*03:01/*DRB*1*16:02	
8	Shangdong, China	DDS-induced DRESS: fever, abnormal liver function tests	A*11:01/A*24:02	3 DDS, 3 DDS-NO CD8
			B*13:01/B*15:25	
			C*03:04/C*04:03	
			*DRB*1*12:02/*DRB*1*15:01	
9	Shangdong, China	DDS-induced DRESS: fever, rash, abnormal liver function tests	A*11:01/A*11:01	3 DDS, 4 DDS-NO CD8
			B*13:01/B*38:02	
			C*03:04/C*07:02	
			*DRB*1*15:01/*DRB*1*15:02	

a CBZ: carbamazepine, DDS: dapsone, DDS-NO: dapsone-nitroso, DRESS: drug reaction with eosinophilia and systemic symptoms, MP: maculopapular exanthema, SMX: sulfamethoxazole, SMX-NO: sulfamethoxazole-nitroso, SJS: Stevens-Johnson syndrome, and TMP: trimethoprim. High-risk HLA markers associated with drug hypersensitivity reactions are in bold.

For the drug-hypersensitive patients, the culprit drugs were CBZ (*n* = 3), SMX-trimethoprim (TMP) (*n* = 2), and DDS (*n* = 2). The clinical history, recruiting site, and HLA genotype are detailed in Table 1. The study protocol was reviewed and approved by the respective local institutional ethics committees—Faculty of Medicine Prince of Songkla University (REC61–216–1–5), Faculty of Medicine Ramathibodi Hospital (MURA2018/650), Liverpool (Adult) Research Ethics Committee (12/NW/0525), and the Ethics Committee of the Shandong Provincial Institute of Dermatology and Venereology (2016ZDJS07A06). All participants gave informed consent. Specifically, 2 CBZ cases were recruited from the University of Liverpool (Liverpool, UK), and one CBZ patient was recruited from the Faculty of Medicine, Prince of Songkla University (Songkhla, Thailand). The two SMX-TMP hypersensitive cases were recruited from the Faculty of Medicine, Ramathibodi Hospital, Mahidol University (Bangkok, Thailand), and were characterized in our previous study.^9^ The two DDS hypersensitive patients were recruited from the Shandong Provincial Institute of Dermatology and Venereology (Jinan, China).^7,10^

### *In Vitro* Generation and Characterization of Monoclonal Drug-Specific T-Cells

The T-cell cloning assay was performed using previously described methods.^7,9–11^ Briefly, the PBMC was incubated with the drug, *in vitro* expanded, and then tested for drug responsiveness before further monoclonal expansion (Supporting Information Methods). A total of 46 *in vitro*-generated drug-specific T-cell clones were included for the TCR analysis (Table 1): 26 CBZ-specific clones, 7 SMX- or SMX-nitroso (NO)-specific clones, and 13 DDS- and DDS-NO-specific clones.^7,9–11^ These clones were characterized for their CD phenotype, drug specificity, and HLA restriction, as detailed in the Supporting Information Methods.

### TCR Sequencing

RNA samples were isolated from monoclonal drug-specific T-cells and quality-checked before library preparation. TCR sequencing libraries were prepared using the SMARTer Human TCR *a*/*b* Profiling Kit (Takara Bio, Japan) according to the manufacturer’s instructions. The prepared TCR libraries were then used to prepare long-read sequencing libraries for single-molecule, real-time (SMRT) sequencing (Pacific Biosciences, United States). A detailed workflow and protocol are described in the Supporting Information Methods.

### Bioinformatic Analysis

Raw sequencing data were processed by the following process: demultiplexing, index checking, V­(D)­J and CDR3 identification, cluster analysis, phylogenetic tree analyses, and CDR3 analyses. Raw data were demultiplexed based on SMRT barcodes using Lima v2.0.0 (available from https://github.com/pacificbiosciences/barcoding). Demultiplexed data for each clone were quality checked before further analysis. TCR characteristics, including VDJ usage, CDR3 characteristics, and clonotype frequency, were identified by using MiXCR. Cluster analysis and phylogenetic tree analysis were done using VDJtools. Alignment and comparison of the CDR3 characteristics were done using WebLogo, T-Coffee, and Jalview.^12^ Full details of bioinformatic analysis are provided in the Supporting Information.

## Results

### Functional Characteristics of Studied T-Cell Clones

Drug responsiveness of all 46 T-cell clones was confirmed through proliferation and interferon-γ release assays. The phenotyping study showed that all CBZ-, SMX-, and SMX-NO-specific clones expressed CD4+ (except one CBZ-specific clone), while all DDS- and DDS-NO-specific clones were predominantly CD8+ (11 out of 13 clones). Cross-reactive responsiveness of CBZ-specific clones was tested by proliferation analyses using CBZ-10,11-epoxide (CBZE). Eight out of 19 tested clones showed a significant proliferation response against CBZE. The HLA restriction of CBZ-specific response in 8 clones was characterized by anti-HLA blocking analyses and HLA mismatch analyses. Three clones were found to be restricted to HLA class II (we were not able to identify specific HLA class II subtypes due to limited cell availability). Two additional clones showed restricted CBZ-mediated responses against HLA-DR (Table 2). The HLA mismatch analyses revealed *HLA*-*DRB*1*07:01 restriction in all 6 tested CBZ-specific clones, and 2 clones also possessed the *HLA*-*DRB*1*04:04 cross-restriction property.

**Table 2:** TCR Characteristics of CBZ-specific CD4+ T-Cell Clones

clone ID number (case-clone)	CBZE cross-reactivity	HLA restriction	TRAV	TRAJ	TRA CDR3	TRBV	TRBJ	TRB CDR3
1–1^a^	strong	HLA II	TRAV38–2DV8	TRAJ33	CAYRSARSNYQLIW	TRBV5–4	TRBJ2–3	CASRSGFIVGDTQYF
82–2^a^, 2–2^a^	no	*DRB*1*07:01, *DRB*1*04:04	TRAV29DV5	TRAJ28	CAASAPGAGSYQLTF	TRBV6–6	TRBJ1–4	CASSPNEKLFF
2–3^a^	no	*DRB*1*07:01	TRAV18–1	TRAJ52	CAPRHEAGTSYGKLTF	TRBV7–9	TRBJ1–2	CASSLALVGYTF
			TRAV8–3	TRAJ49	CAVAPNTGNQFYF	-	-	-
2–4^a^	good	*DRB*1*07:01	TRAV13–1	TRAJ20	CAASNDYKLSF	TRBV12–4	TRBJ2–7	CASSFGGSGYEQYF
2–5^a^, 2–6^a^	strong	HLA II	TRAV13–1	TRAJ20	CAASNDYKLSF	TRBV12–4	TRBJ2–1	CASSFGGRSYEQFF
2–7^a^, 2–8^a^	strong	*DRB*1*07:01	TRAV13–1	TRAJ20	CAASNDYKLSF	TRBV12–4	TRBJ2–7	CASSFGGSSYEQYF
3–1^b^	n.d.	n.d.	TRAV12–2	TRAJ53	CAVNMRNSGGSNYKLTF	TRBV7–2	TRBJ2–3	CASSSVGLNPQYF
3–2^b^ ^,^ ^c^	good	n.d.	TRAV26–1	TRAJ30	CIVIGDDKIIF	TRBV12–4	TRBJ2–1	CASSFQLIQNEQFF
3–3^b^	no	n.d.	TRAV12–2	TRAJ26	CAALYNYGQNFVF	TRBV27	TRBJ2–7	CASSLVLGQGSYEQYF
3–4^b^, 3–5^b^	n.d.	HLA-DR	TRAV17	TRAJ54	CATTDLIQGAQKLVF	TRBV7–3	TRBJ2–1	CASSQGARRTTEQFF
			TRAV26–1	TRAJ58	CIVSYTPKETSGSRLTF	-	-	-
4–1^b^, 4–2^b^	strong	n.d.	TRAV12–3	TRAJ52	CAMSYAGGTSYGKLTF	TRBV10–3	TRBJ2–3	CAIRDRGQGSTDTQYF
			TRAV25	TRAJ48	CAGGSNFGNEKLTF	-	-	-
4–3^b^	no	HLA-DR	TRAV21	TRAJ42	CAVGGSQGNLIF	TRBV5–1	TRBJ1–6	CASSLRTGENSPLHF
4–4^b^, 4–5^b^, 4–6^b^, 4–7^b^	no	HLA II	TRAV1–2	TRAJ38	CAVSGHAGNNRKLIW	TRBV12–4	TRBJ1–2	CASTRQGVDYGYTF
4–8^b^	n.d.	n.d.	TRAV36DV7	TRAJ33	CAVGNSNYQLIW	TRBV12–4	TRBJ2–6	CASSFYRVAGANVLTF
4–9^b^	n.d.	n.d.	TRAV8–6	TRAJ57	CAVIRSEKLVF	TRBV7–8	TRBJ2–7	CASSLGLTYEQYF
4–10^b^, 5–1^b^	weak	n.d.	TRAV8–3	TRAJ49	CAVGAQDTGNQFYF	TRBV10–3	TRBJ2–7	CATAGLSSYEQYF
			-	-	-	TRBV12–5	TRBJ1–5	CARVKDQPQHF
5–2^b^	n.d.	n.d.	TRAV38–2DV8	TRAJ57	CAFRGGSEKLVF	TRBV6–5	TRBJ2–1	CASSLQGRRYNEQFF
			TRAV17	TRAJ23	CATDNQGGKLIF	TRBV20–1	TRBJ1–5	CSASRGRTNQPQHF

a Clones were derived from healthy donors with high-risk alleles.

b Clones derived from hypersensitive patients.

c All clones expressed a CD4+ phenotype except clone number 3–2 expressed CD8+ phenotype. n.d.: not determined.

For T-cell clones generated from SMX-TMP hypersensitive patients, all seven clones showed the CD4+ phenotype and an SMX-NO-specific response. A cross-reactive response was identified in two clones: one against SMX and the other against DDS-NO. HLA restriction tested in 2 clones revealed HLA class II restriction (Table 3). No HLA mismatch analyses were done. All 13 DSS- and DSS-NO-specific T-cell clones showed a CD8+ phenotype in proliferative and IFN-γ release analyses (Table 4). The HLA mismatch analyses showed promiscuous HLA restriction among DSS- and DDS-NO-specific T-cell clones.

**Table 3:** TCR Characteristics of SMX- and SMX-NO-specific CD4+ T-Cell Clones^a^

clone ID number (case-clone)	specificity	HLA restriction	TRAV	TRAJ	TRA CDR3	TRBV	TRBJ	TRB CDR3
6–1	SMX, SMX-NO	n.d.	TRAV38–1	TRAJ22	CAFMKPSTSGSARQLTF	TRBV5–1	TRBJ2–1	CASSLFLAGANEQFF
6–2	SMX-NO	HLA class II	TRAV13–1	TRAJ29	CAASIPGNTPLVF	TRBV20–1	TRBJ1–2	CSARGGAGGYTF
			TRAV13–2	TRAJ4	CAENMAGGYNKLIF	-	-	-
6–3	SMX-NO	HLA class II	TRAV4	TRAJ31	CLVGYNARLMF	TRBV20–1	TRBJ2–1	CSASRREAYNEQFF
7–1	SMX-NO, DDS-NO	n.d.	TRAV12–3	TRAJ34	CAANTDKLIF	TRBV20–1	TRBJ2–7	CSASRRPYEQYF
7–2	SMX-NO	n.d.	TRAV13–2	TRAJ6	CAERGGGSYIPTF	TRBV6–2	TRBJ2–1	CASSYLAGGQEQFF
7–3, 7–4	SMX-NO	n.d.	TRAV12–1	TRAJ42	CAVNRNYGGSQGNLIF	TRBV9	TRBJ2–1	CASSVAGGYNEQFF

a All clones were derived from hypersensitive patients. SMX, sulfamethoxazole; SMX-NO, nitroso-sulfamethoxazole; and DDS-NO, nitroso-dapsone.

**Table 4:** TCR Characteristics of DDS- and DDS-NO-specific CD8+ T-Cell Clones^a^

clone ID number (case-clone)	TRAV	TRAJ	TRA CDR3	TRBV	TRBJ	TRB CDR3
DDS-specific *HLA*-B*15:25-restricted clones from patient						
8–1	TRAV12–2	TRAJ16	CAVMGFSDGQKLLF	TRBV6–1	TRBJ1–2	CASREDRGGAGYTF
8–2	TRAV10	TRAJ54	CVVSAILGAQKLVF	TRBV6–5	TRBJ2–6	CASSYSAKTSGANVLTF
	TRAV35	TRAV18	CAGQQVRGSTLGRLYF	TRBV12–3	TRBJ2–3	CASRLGQGASNF
8–3	TRAV10	TRAJ54	CVVSAILGAQKLVF	TRBV6–5	TRBJ2–6	CASSYSAKTSGANVLTF
DDS-NO-specific *HLA*-B*13:01-restricted clones from patient						
8–4, 8–5	TRAV24	TRAJ42	CAFHGGSQGNLIF	TRBV9	TRBV1–1	CASSVDGLNTEAFF
8–6	TRAV1–1	TRAJ26	CAPSYGQNFVF	TRBV3–1	TRBJ2–7	CASSQDREGSYEQYF
DDS-specific *HLA*-B*13:01-restricted clones from patient						
9–1	TRAV19	TRAJ45	CALINSGGGADGLTF	TRBV5–4	TRBJ2–7	CASSTSLGEVGYEQYF
	TRAV25	TRAJ48	CAGSNFGNEKLTF	TRBV10–1	TRBJ2–1	CASSEATGTGGYNEQFF
9–2	TRAV38–2DV8	TRAJ28	CASAGSYQLTF	TRBV7–9	TRBJ2–7	CASSSTAGRPYEQYF
9–3	TRAV17	TRAJ52	CATLGGTSYGKLTF	TRBV27	TRBJ1–1	CASSLEGQRAFF
	TRAV5	TRAJ32	CAEAYGGATNKLIF	TRBV28	TRBJ2–1	CASSHRGLVSSYNEQFF
DDS-NO-specific *HLA*-B*13:01-rrestricted clones from patient						
9–4, 9–5	TRAV14DV4	TRAJ50	CAMGTSYDKVIF	TRBV7–9	TRBJ2–5	CASSSNTEEQETQYF
DDS-NO-specific HLA Class II-restricted clones from patient						
9–6, 9–7	TRAV4	TRAJ33	CLVGYSNYQLIW	TRBV11–2	TRBJ2–2	CASSQRTGNTGELFF

a DDS, dapsone; DDS-NO, dapsone-nitroso.

### Demultiplexing and Characterization of TCR α/β Transcripts

Sequencing reads were successfully demultiplexed, extracted, and split for each T cell sample. All SMRT barcodes and Takara Bio primer barcodes were correctly identified in the demultiplexed data, as assigned during library preparation. The average total sequencing reads and successfully aligned sequencing reads from the demultiplexed data were 12,535 reads and 6846 reads, respectively.

The VDJ usage of each T-cell was identified, showing a prominent pair of productive TCR α/β chains for most clones (Figure 1). Among 46 tested samples, 33 displayed a single dominant pair of prominent TCR α/β transcripts (Figure 1A), while 13 samples showed more than one productive TCR α/β transcripts (Figure 1B–D). Among samples with multiple TCR α/β transcripts, 7 showed two TCRα transcripts, 2 showed two TCRβ transcripts, and 4 showed two transcripts of both TCRα and TCRβ.

**Figure 1:**
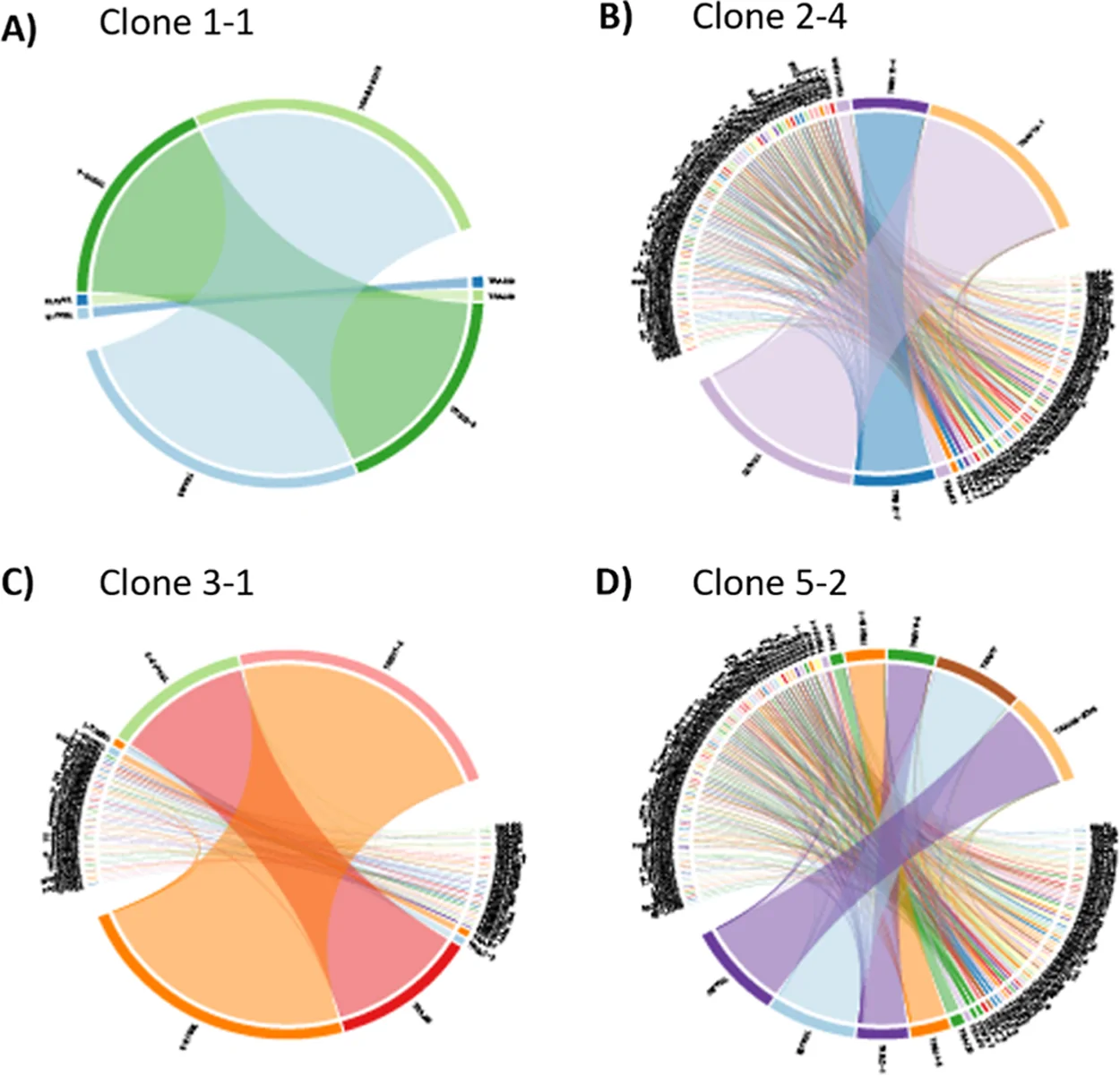
TCR characteristics of T-cell clones. Four representative clones with different TCR pairing results are shown. (A) Clone 1–1 showed a pair of prominent TCR α/β chains (thick blue bar and thick green bar, respectively) with 2 minor noise reads (thin lines). (B,C) Clone 2–4 and 3–1 showed a pair of prominent TCR α/β chains (thick bars) with multiple noise reads (thin lines). (D) Clone 5–2 showed 2 pairs of productive TCR α/β chains (4 thick bars) with multiple minor noise reads (thin lines).

### Preferential TCRβ Usage in CD4+ Drug-Specific T-Cell Clones

The identification and comparison of VDJ usage and CDR3 length revealed preferential TCRβ usage among drug-specific T-cells. We observed a skewed CDR3β length distribution and preferential *TRBJ* and *TRBV* gene usage (Figure 2). This trend was not observed for *TRAJ*, *TRAV*, or CDR3α. The length of CDR3α was evenly distributed between 11 and 16 residues (Figure 2A), while the length of CDR3β prominently clustered around 14 and 15 residues (Figure 2B).

**Figure 2:**
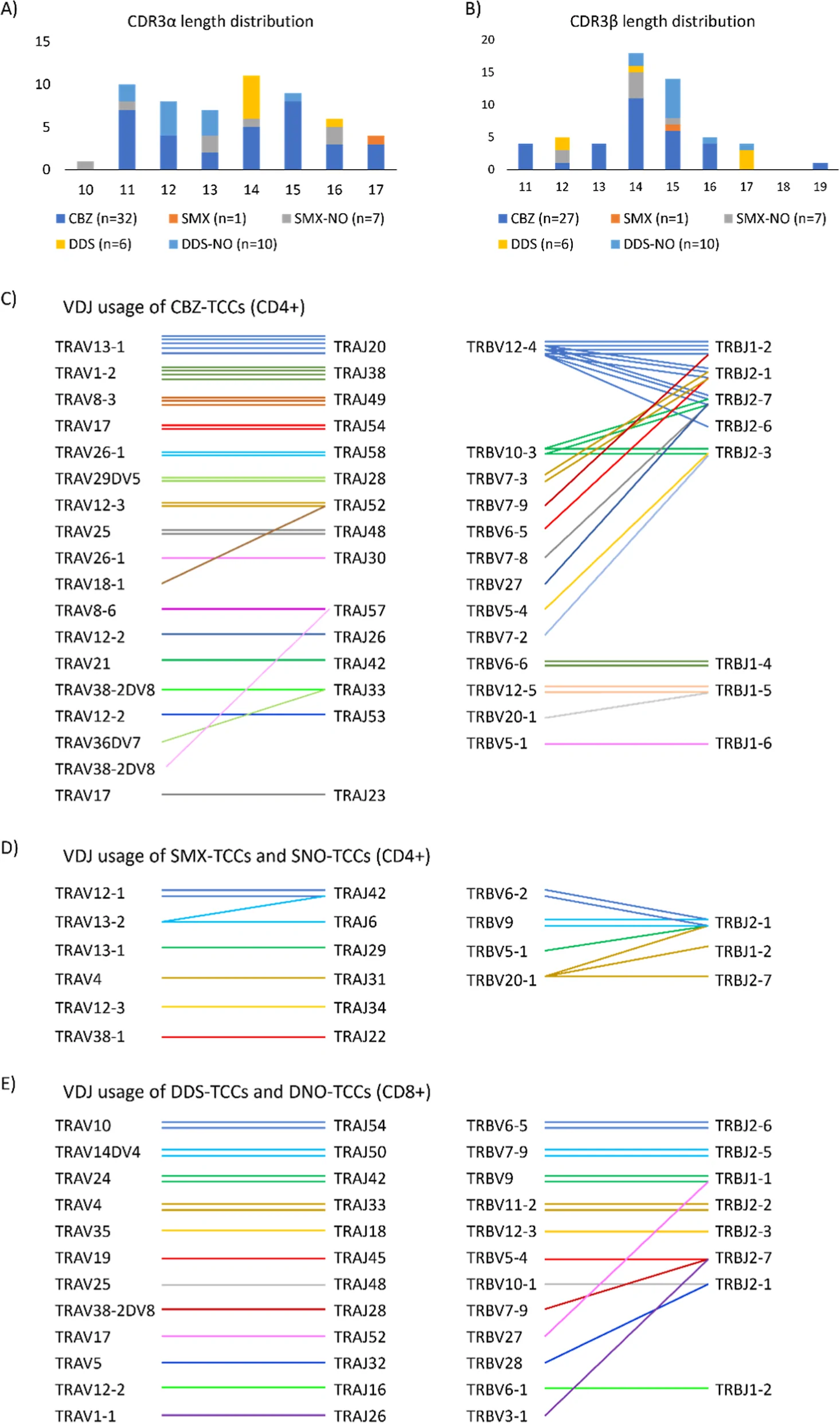
CDR3 and V­(D)­J usage of drug-specific T-cell clones. (A,B) Graphs showing the count of TCR chains with specific CDR3α and CDR3β lengths. (C–E) Each line represents V­(D)­J pairing of one TCR chain. The left panel shows TRA usage, and the right panel shows TRB usage. CBZ-, SMX-, and SMX-NO-specific T-cell clones showed more preferential usage than DDS- and DDS-NO-specific T-cell clones. CBZ: carbamazepine, DDS: dapsone, DNO: nitroso-dapsone, SMX: sulfamethoxazole, SNO: nitroso-sulfamethoxazole, and TCC: T-cell clone.

Shared *TRBJ* or *TRBV* genes were observed among CD4+ CBZ-, SMX-, and SMX-NO-specific T-cell clones (Figure 2C,D and Tables 2 and 3) but not CD8+ DDS- and DDS-NO-specific T-cell clones (Figure 2E and Table 4). Among 31 CD4+ CBZ-specific T-cell clones, *TRBV*12–4 was expressed in 11 clones (35%), while *TRBJ*1–2, *TRBJ*2–1, and *TRBJ*2–7 were expressed in 5, 6, and 7 clones, respectively. Consistently, 3 out of 8 CD4+ SMX- and SMX-NO-specific T-cell clones expressed *TRBV*20–1, and 6 out of 8 clones expressed *TRBJ*2–1. However, no preferential TCRβ usage was observed among CD8+ DDS- and DDS-NO-specific T-cell clones (Figure 2E).

### Clustering and Phylogenetic Analysis of T-Cell Clones

Cluster analysis performed using VDJtools showed a loose cluster with similar clonal functions, but phylogenetic analysis indicated that most clones were not closely related (Figure 3). Only five clones from a healthy donor carrying *HLA*-B*15:02 (case 2; Tables 2 and 3) grouped together in both cluster and phylogenetic analyses. These clones shared CBZE cross-reactivity and *HLA*-*DRB*1*07:01 restriction property (Figure 3 and Table 2). Additionally, multiple identical clones were identified based on their identical VDJ gene usage and CDR3 sequences (Figure 3 and Tables 2–4). SMRT and Takara Bio barcodes confirmed that these TCRs originated from different samples, which ruled out contamination.

**Figure 3:**
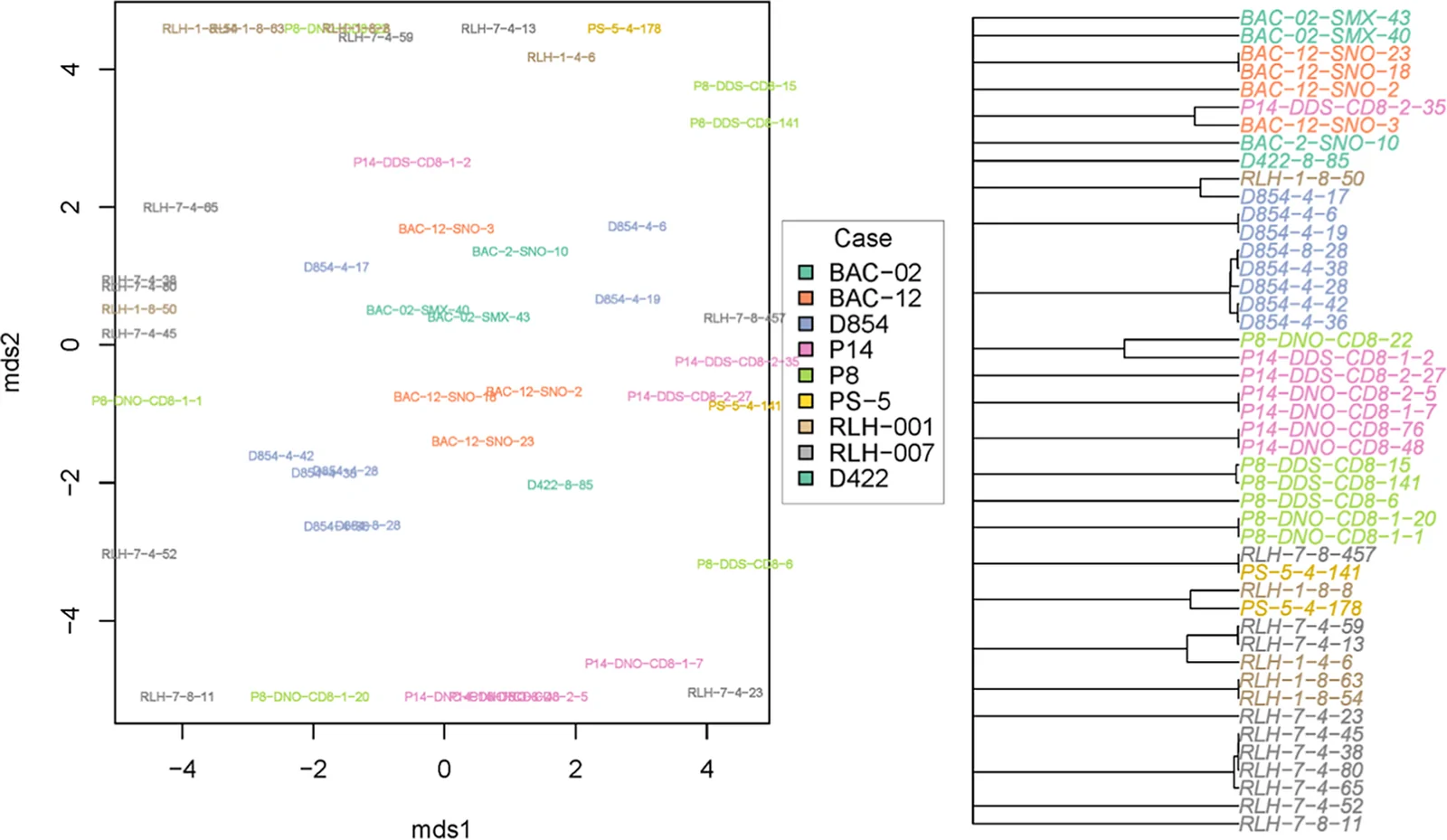
Multidimensional scaling plot and phylogenetic tree analysis of drug-specific T-cells based on pairwise similarities of TCRs. BAC-02 and BAC-12 clones were derived from patients with sulfamethoxazole-induced hypersensitivity reactions (case nos. 6 and 7, respectively). D422 and D854 clones were derived for healthy donors with high-risk alleles for carbamazepine hypersensitivity reactions (case nos. 1 and 2, respectively). RLH-001, RLH-007, and PS-5 clones were from patients with carbamazepine-induced hypersensitive reactions (case nos. 3, 4, 5, respectively). P8 and P14 clones were derived from patients with dapsone-induced hypersensitivity reactions (case no.8, 9, respectively).

### Shared but Nonrestrictive CDR3 Characteristics in CBZ-Specific T-Cell Clones

Figure 4 shows sequence alignment results, using WebLogo, of CDR3α and CDR3β of similar length—CDR3α with 11 and 15 residues (*n* = 7 and 8, respectively), and CDR3β with 14 and 15 residues (*n* = 9 and 5, respectively). A high proportion of lysine–leucine (KL) residues was observed at positions 8–9 of CDR3α with 11 residues (Figure 4A) and positions 12–13 for the CDR3α with 15 residues (Figure 4B). For the CDR3β with 14 residues, a glycine (G) residue at position 7 and a tyrosine (Y) residue at position 10 were conserved (Figure 4C). However, comparison of CDR3β with 15 residues showed no clear conservation, partly due to the limited number of CDR3s. No other residue was identified as conserved, except the cystine-alanine (CA) residue at positions 1–2 and a phenylalanine (F) residue at the CDR3 C-terminus (Figure 4D). Furthermore, analysis of nonduplicated CDR3α/β from CBZ-specific clones with different lengths using T-Coffee tool and Jalview confirmed the preserved KL residue on CDR3α, while CDR3β showed a lesser degree of conservation (Figure 4E). The comparison of CDR3β revealed a shared YEQYF residue in 5 out of 16 CBZ-specific T-cell clones, correlating with the preferential use of *TRBJ*2–7 (Table 4). However, these shared residues were not conserved among other T-cell clones.

**Figure 4:**
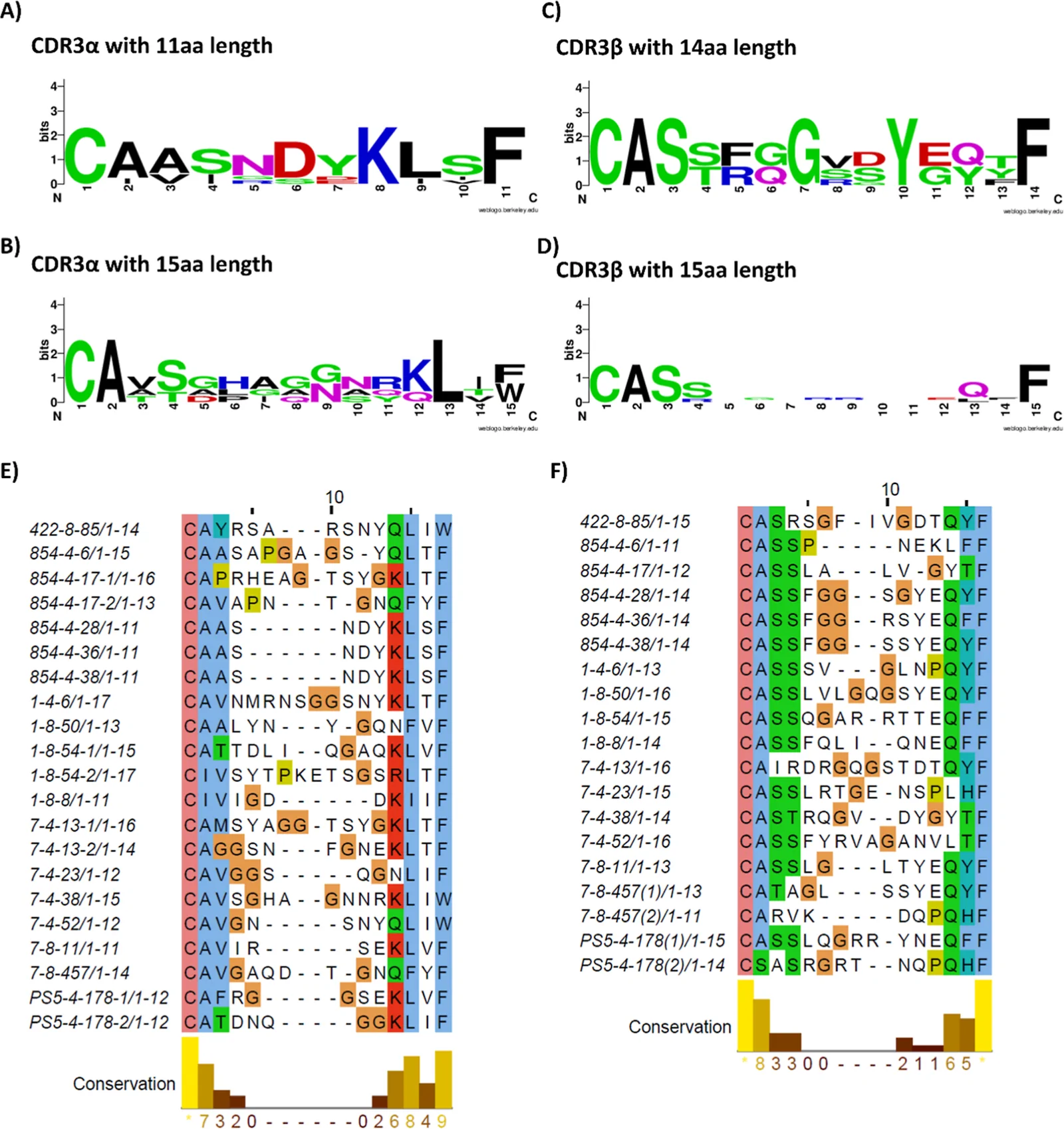
CDR3 alignment of carbamazepine-specific T-cell clones. (A–D) Alignment based on the WebLogo online tool revealed the CDR3α with 11- and 15-residue length and CDR3β with 14- and 15-residue length. Each letter stack represents each position of amino acid residue. The letter height represents the conservation of the residue. (E,F) CDR3α and CDR3β alignment using Dialign tool showed no conserved CDR3 usage besides the initial and end residues of the CDR3. Only nonduplicated clones were included in this analysis. aa: amino acid.

## No Association between CDR3 Residue and Drug-Specific T-Cell Responsiveness

Figure 5 shows the sequence alignment of CDR3 of CBZ-specific T-cell clones with similar functional characteristics to evaluate possible structure–function association. The CDR3s of CBZ-specific T-cell clones with and without CBZE cross-reactivity (T-cell clones with a weak cross-reactive response were excluded) were aligned. The CDR3α alignment showed no conserved residue among T-cell clones with CBZE cross-reactive property (Figure 5A). In contrast, the CDR3β alignment revealed shared glycine (G) residues at positions 6–7 in all 5 clones with CBZE cross-reactivity and in one noncross-reactive T-cell clone (Figure 5B,C). However, in addition to this conserved glycine among T-cell clones with CBZE cross-reactive property, no other conserved residue was observed among clones with *HLA*-*DRB*1*07:01, HLA-DR, or HLA class II restriction property (Figure 5D,E).

**Figure 5:**
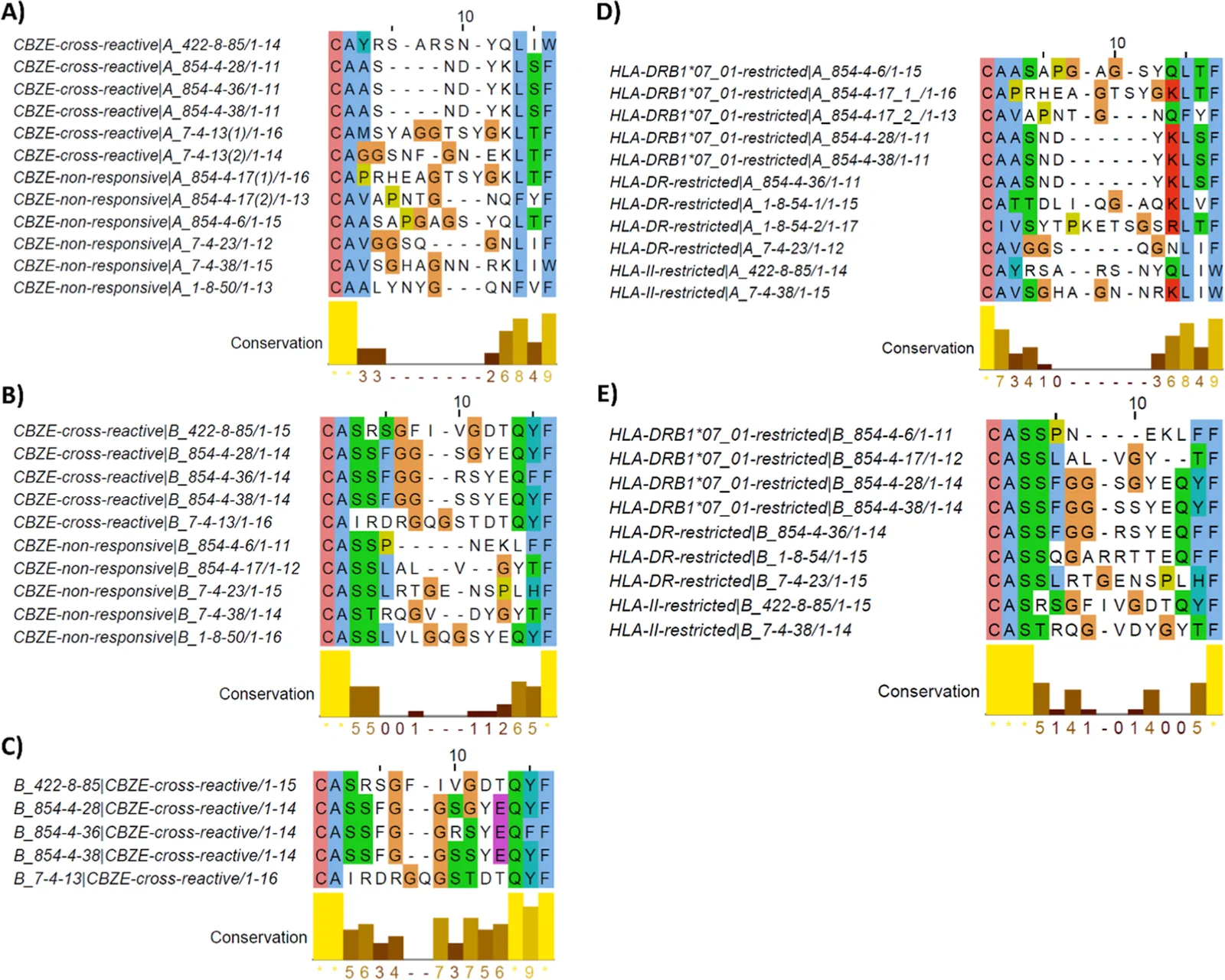
CDR3 comparison of CBZ-specific T-cell clones based on functional characteristics. (A,B) CDR3α and CDR3β were aligned and compared based on their carbamazepine-10,11-epoxide (CBZE) cross-reactivity. (C) Subgroup analysis among CBZE-cross-reactive clones showed conserved alignment of glycine residues. (D,E) CDR3 comparison based on HLA restriction properties showed no conserved residue was observed among clones.

### Shared CDR3 Characteristics in CD4+ SMX- and SMX-NO-Specific T-Cell Clones, but Not CD8+ DDS- and DDS-NO-Specific T-Cell Clones

CDR3 alignment of SMX- and SMX-NO-specific CD4+ T-cells revealed a shared NEQFF residue on the J end of CDR3β, which correlated to *TRBJ*2–1 usage (Table 4 and Figure 6). This residue was identified in 4 out of 7 clones, including one SMX-specific clone with SMX-NO cross-reactive response. Additionally, glycine residues were shared but not strictly conserved at positions 10–11 of CDR3α (Figure 6A,B).

**Figure 6:**
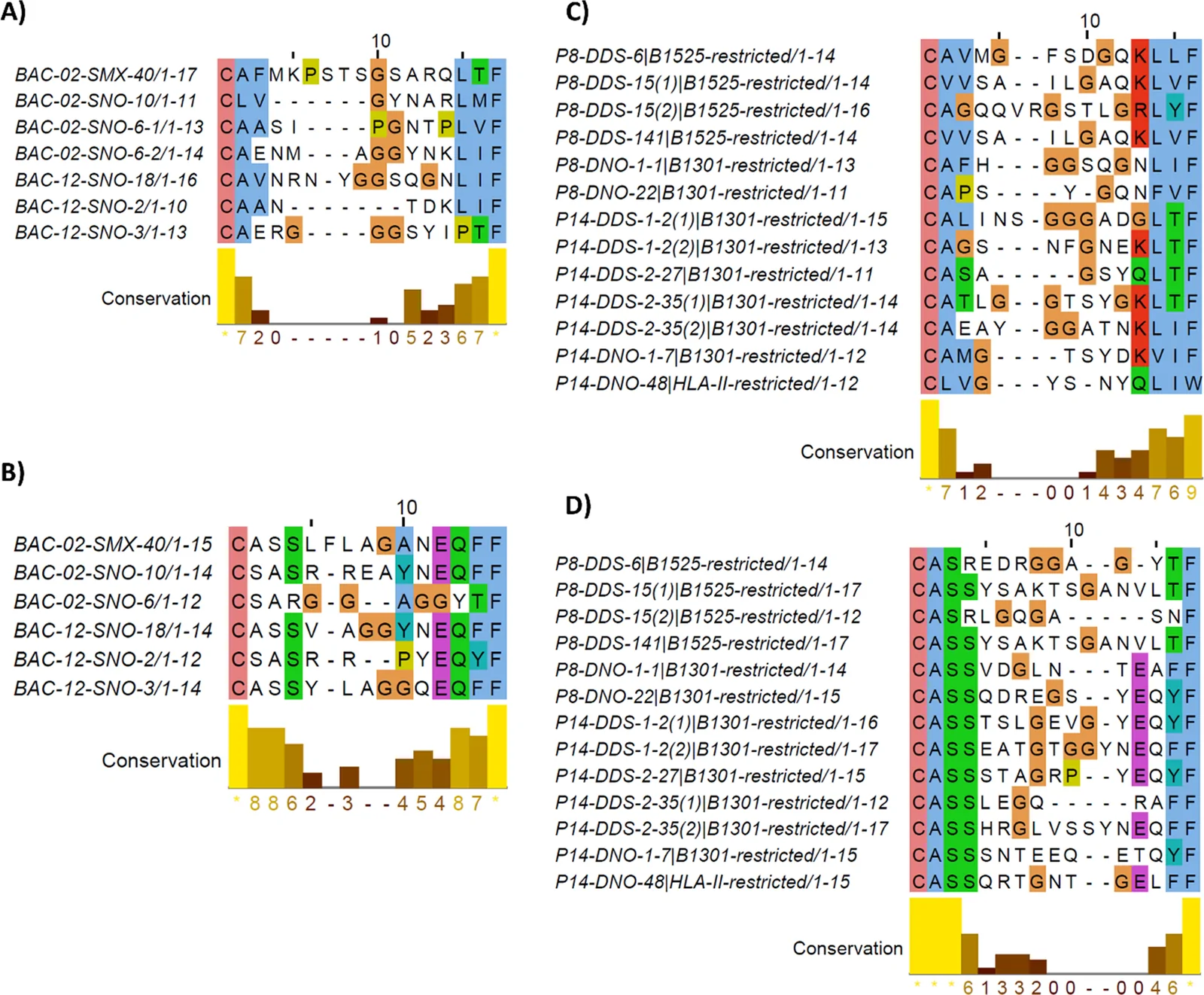
CDR3 alignment of sulfamethoxazole-, sulfamethoxazole-nitroso-, dapsone-, and dapsone-nitroso-specific T-cell clones. A comparison of CDR3α (A) and CDR3β (B) of sulfamethoxazole- and sulfamethoxazole-nitroso-specific T-cell clones showed no conserved CDR3 residue among clones. Only glutamic acid–glutamine (EQ, purple and green) residues were shared in most, but not all, of CDR3β. A comparison of CDR3α (C) and CDR3β (D) of dapsone- and DDS-NO-specific T-cell clones. Showed no conserved residue.

For CD8+ DDS- and DDS-NO-specific T-cell clones, the TCR sequences were consistent with previously published data on the same samples.^7^ Minor TCRα and TCRβ transcripts were identified in 3 clones (Table 4). The CDR3 alignment of DDS- and DDS-NO-specific T-cell clones showed no shared characteristics or conserved residues among the clones (Figure 6C,D).

## Discussion

In this study, we characterized the TCR expressed by drug-specific T-cells derived from drug-naïve healthy donors and hypersensitive patients and evaluated the possible correlation between CDR3 residues and functional characteristics of the clones. An oligoclonal nature of drug-specific TCRs was identified without any possible correlation between CDR3 and drug response characteristics. The expressed *TRBV* from CBZ-specific CD4+ T-cells revealed herein were consistent with the previously identified TCR Vβ phenotypes^7,11^ and also consistent with those expressed by CD8+ CBZ-specific T-cells in previous reports.^3,4^ A shared *TRBJ*2–1 usage was also identified among CBZ-, SMX-, and SMX-NO-specific T-cell clones. This suggested a possible shared antigen recognition site between CBZ and SMX. The severe cutaneous reactions induced by these two drugs were similarly reported to be associated with *HLA*-B*15:02.^13,14^ This preferential *TRBJ*2–1 usage suggests a conserved recognition mechanism where *TRBJ*2–1-encoded CDR3β enables interactions of structurally distinct drug molecules onto HLA-B*15:02 molecule via overlapping TCR binding capability.

A comparison of drug-specific TCRs between studies showed wide heterogeneity across the literature (Table 5). This suggests that the CDR3 alone is unlikely to be a key determining factor for drug-mediated T-cell response and indicates drug molecules drive polyclonal T-cells via a wide variety of HLA-TCR complexes. As CDR1 or CDR2 are mostly conserved, their contribution to the TCR-drug-HLA interaction might require an alternative approach besides the characterization of TCR diversity. The antigen specificity and drug-mediated response could be defined by the massive numerical possibility of both α chain and β chains along with the specific cellular characteristic of T-cells that possess the drug-specific TCRs.^15,16^ Additionally, a recent multiomic single-cell study also reported a sharing of CD8+ TCR identified in affected skin and blister fluid of SJS patients in which the -YEQYF residue of CDR3β was shared among dominant clonotypes.^17^ This also supports the mechanistic hypothesis that CD4+ and CD8+ T-cells shared drug-specific responsiveness via very similar TCR characteristics.^11^

**Table 5:** Comparison of Drug-Specific TCRβ Reported in This Study and Previous Studies^a^

T-cell population and sample	culprit drug	HLA marker	drug specificity testing	common TRBV usage	common TRBJ usage	example of common CDR3β
CD4+ T-cell clones *in vitro* generated from donors and patients (this study)	carbamazepine	B*15:02, B*57:01, A*31:01	CBZ-mediated proliferation, IFN-γ secretion	TRBV12–4	TRBJ1–2, TRBJ2–1, TRBJ2–7	CASTRQGVDYGYTF
						CASSFGGSGYEQYF
	sulfamethoxazole	B*13:01	drug-mediated proliferation	TRBV6–2, TRBV9, TRBV20–1	TRBJ2–1	CASSVAGGYNEQFF
CD8+ T-cell clones *in vitro* generated from donors and patients (this study)	dapsone	B*13:01	drug-mediated proliferation	TRBV9	TRBJ1–1	CASSVDGLNTEAFF
					TRBJ2–7	CASSTSLGEVGYEQYF
T-cells (phenotype not available) from active patients’ PBMC^18^	carbamazepine	NA	relative frequency of V and J gene compared to healthy control	TRBV20–1, TRBV12–3, TRBV6–5	TRBJ1–3	CAWSQERQFF CASSPSNSPLHF
					TRBJ2–5	
					TRBJ1–5	
T-cells (mainly CD8) from patients’ PBMC and blister cells^3^	carbamazepine	B*15:02	relative frequency of V and J gene, mouse model	TRBV12–4	TRBJ2–2, TRBJ2–7	ASSLAGELF
						ASSPSDRSSYEQY
						ASSWDPTIY
CD8+ T-cells *in vitro* expanded from donors and recovered patients’ PBMC^4^	carbamazepine	with and without B*15:02	IFN-γ expression	TRBV27	TRBJ1–1, TRBJ2–3, TRBJ2–7	CASRAGGNTEAFF
						CASSSLSGGWPDTQYF
						CASSQEGRGRYEQYF
						CASSSLSGGWPDTQYF
CD8+ T-cells from affected skin and blister fluids of SJS patients^17^	carbamazepine, allopurinol, lamotrigine, sulfamethoxazole-trimethoprim, nevirapine, pyrazinamide	A*31:01, B*15:21, B*58:01, B*38:01, C*04:01	single-cell multiomic analysis	TRBV27	TRBJ2–7	CASSPDRGGYEQYF
						CASSQDRGGYEQYF
T-cells (both CD4+ and CD8+) from an active patient^8^	Sulfamethoxazole	NA	clone frequency and transcription profile	NA	NA	strikingly diverse TCR clonotype
T-cells (CD4+ and CD8+) from blister cells and *in vitro* expanded culture from patients^5^	allopurinol	with and without B*58:01	clone frequency, T-cell activation assay	TRBV29–1 TRBV3–1	TRBJ2–5 TRBJ1–3	SVLIEGTQY
						SVERLAGQETQY
						ASSQDLTGNTIY
T-cells (CD8+) from *in vitro* expanded culture from donors and patients^6^	allopurinol	with B*58:01	IFN-γ, TNF-α expression	TRBV20–1	TRBJ1–4	CSARVYTEGYEKLFF

a CBZ: carbamazepine, NA: data not available, PBMC: peripheral blood mononuclear cell, and SJS: Stevens-Johnson syndrome.

The polyclonal patterns reflect antigen identity. Varied drugs generate distinct antigenic epitopes—providing an insight on observed TCR repertoire diversity. Furthermore, while distinct cutaneous phenotypes might similarly be driven by polyclonal T-cell response, current evidence remains insufficient to establish a conclusion of this link, warranting further investigation into this prospective mechanism.

In terms of structure–function correlation, a conserved glycine on CDR3β was observed among CBZ-specific T-cell clones displaying CBZE cross-reactivity. However, no other correlation was identified between TCR characteristics and drug-specific response. Hypothetically, it could indicate that drug responsiveness behavior is linked to the molecular conformation of the HLA antigen-presenting surface rather than a CDR3 residue. It is also important to highlight that, besides TCRs, there could also be some other factors that could impact T-cell function, for example, cellular epigenetic changes, T-cell exhaustion, coinhibitory, and costimulatory receptors. Thus, further validation (e.g., an alignment on full receptor sequence, docking simulation, or crystal structure analysis) and animal model studies will be important to reveal clearer details of the molecular interaction between drug and immune receptors. In particular, molecular docking with all-atom molecular dynamics simulations and binding free energy calculations will provide a more accurate characterization of the TCR-pMHC interface by capturing the dynamic behavior and energetic effects of specific mutations.

In addition to TCR, the presented HLA and peptides that involve drug-antigen recognition and immune activation are also crucial. Previously, *in vitro* characterization of drug-presenting HLA showed several HLA molecules that were restricted to drug presentation, including *HLA*-*DRB*1*07:01-restricted CBZ recognition,^11^ HLA class II-restricted SMX and SMX-NO recognition,^9^ and *HLA*-B*15:25-restricted DDS and DDS-NO recognition.^7^ In terms of immunopeptidomics, this present study has a limitation on the insights into presenting peptides. In the initiation of antigen recognition, T-cells recognize peptides rather than a drug in cases of the hapten model and might be critical in the pharmacological interaction of drugs with the immune receptors (p–i) model.^19^

Despite extensive investigations, using TCR sequencing to predict or prevent drug hypersensitivity reactions remains beyond our current technological capability. Although the identification of drug-specific TCR was proposed as a potential biomarker for SJS/TEN as early as 2011, its clinical implementation has yet to be proven.^2,20^ The recent advancement of single-cell sequencing has led to more comprehensive investigations and theoretical approaches for TCR studies.^3,15,17^ However, a key challenge for the practical use of TCRs as biomarkers lies in their diversity and dynamics. TCR repertoires are known to differ based on geographical location, age, sex, and recent infections, making the evaluation of TCR difficult for *in vivo* study, *in vitro* testing, and *in silico* prediction.^19,21^ Previous studies have reported an association between the expansion of a dominant T-cell clonotype (or a decrease in overall T-cell clonal diversity) and the severity of drug hypersensitivity reactions.^18,22^ As a step toward this, the set of drug-specific TCRs and their functional characteristics described in this study can serve as a foundation for further molecular modeling to predict drug-TCR-HLA interactions. Ultimately, expanding the TCR data set with corresponding functional data will likely require machine learning approaches for more extensive analyses and a better understanding of this complex puzzle.

To summarize, this study has revealed that there is wide heterogeneity in TCRs expressed by drug-specific T-cells. A major limitation of the study was the availability of hypersensitive patients’ PBMC when the reactions were active. Nevertheless, our data suggest that it will be difficult to predict drug hypersensitivity reactions based on TCR information alone since the extent of TCR heterogeneity and the functional correlation are obscure. Future experiments on the distinct CDR3 T-cell clones emerge after sorting cells with HLA-matched dimers could reveal an emergence of distinct CDR3 sequences.

## Supplementary Material



## Notes

Generation and characterization of drug-specific T-cell clones, RNA isolation, preparation of TCR sequencing libraries and TCR sequencing, and bioinformatic analyses (PDF)

## Abbreviations

CBZ: carbamazepine
CD: cluster of differentiation
CDR3: complementary determining region 3
DDS: dapsone
DRESS: drug reaction with eosinophilia and systemic symptoms
HLA: human leukocyte antigen
MP: maculopapular exanthema
NO: nitroso
PBMC: peripheral blood mononuclear cells
SJS: Stevens-Johnson syndrome
SMX: sulfamethoxazole
TCR: T-cell receptor
TMP: trimethoprim
VDJ: variable-diversity-joining
