# Supplementary material for: Shared and Distinct T‑Cell Receptor Characteristics of CD4+ and CD8+ Drug-Specific T‑Cells In Vitro Generated from Healthy Donors and Hypersensitive Patients
Source: Chem Res Toxicol. 2026 Aug 18;39(9):1735–47. doi: 10.1021/acs.chemrestox.6c00152 (PMC13599470; doi:10.1021/acs.chemrestox.6c00152)
Supplement: Supplementary file 1 [file tx6c00152_si_001.pdf]

Supporting Information

Shared and distinct T-cell receptor characteristics of CD4+ and CD8+ drug-specific T-cells *in vitro* generated from healthy donors and hypersensitive patients

Kanoot Jaruthamsophon [1,2], Paul J Thomson [2], Jirawat Pratoomwun [2,3,4,5], Qing Zhao [2,6], Mubarak Almutairi [2,7], Antida Sangiemchoey [8], Araya Yeunyongviwat [9], Kanitpong Phabphal [10], Hong Liu [6], Furen Zhang [6], Yonghu Sun [6], Chonlaphat Sukasem [2,3,4,11], Munir Pirmohamed [2], Dean J Naisbitt [2,]*

AUTHOR ADDRESS

1 Department of Pathology, Faculty of Medicine, Prince of Songkla University, Songkhla, Thailand 90110

2 Centre for Drug Safety Science, Department of Pharmacology and Therapeutics, Institute of Systems, Molecular and Integrative Biology, University of Liverpool, Liverpool L69 3GE, U.K.

3 Division of Pharmacogenomics and Personalized Medicine, Department of Pathology, Faculty of Medicine Ramathibodi Hospital, Mahidol University, Bangkok 10400, Thailand.

4 Laboratory for Pharmacogenomics, Somdech Phra Debaratana Medical Center (SDMC), Ramathibodi Hospital, Bangkok 10400, Thailand.

5 Department of Clinical Chemistry, Faculty of Medical Technology, Huachiew Chalermprakiet University, Samut Prakan, 10540, Thailand.

6 Shandong Provincial Hospital for Skin Diseases & Shandong Provincial Institute of Dermatology and Venereology, Shandong First Medical University & Shandong Academy of Medical Sciences, Jinan, 250022, China

7. Department of Medical Laboratory Sciences, College of Applied Medical Sciences in Alquwayiyah, Shaqra University, Shaqra, Riyadh Province, 15526, Saudi Arabia

8 Division of Pharmacy, Songklanagarind Hospital, Faculty of Medicine, Prince of Songkla University, Songkhla 90110, Thailand

9 Department of Pediatrics, Faculty of Medicine, Prince of Songkla University, Songkhla, 90110, Thailand

10 Department of Internal Medicine, Faculty of Medicine, Prince of Songkla University, 90110, Songkhla, Thailand

11 Faculty of Pharmaceutical Sciences, Burapha University, Saensuk, Mueang, Chonburi, 20131, Thailand.

***Corresponding author email:** DJN: dnes@liverpool.ac.uk

Table of Contents

Contents

1 T-cell cloning and specificity testing of T-cell clones S4
2 Cellular characterization of drug-specific T-cell clones S4
3 HLA restriction assays S4
4 RNA isolation for TCR sequencing S5
5 Library preparation of TCR transcript S7
6 Long-read sequencing of the TCR libraries S9
7 Bioinformatic analysis S11
7.1 Demultiplexing of the pooled sequencing data S11
7.2 V(D)J segment and CDR3 identification S12
7.3 Cluster analysis and phylogenetic tree analysis S12
7.4 CDR3 alignment S12
7.5 Comparison of physicochemical properties of the CDR3s S12
8 Supplementary references S14

Supplementary Methods

1 T-cell cloning and specificity testing of T-cell clones

T-cell clones (TCC) were generated by T-cell cloning methodologies as previously described. [1] Firstly, CBZ-incubated bulk culture was generated by incubating 1×10^6 peripheral blood mononuclear cells (PBMC) with CBZ (25 and 50 μg/ml), and fed with IL-2 supplemented R9 medium on day 6 and 9. The R9 medium is comprised of RPMI 1640 (Sigma), 10% pooled human blood type AB serum (Innovative Research, USA), 25 mM HEPES buffer (Sigma), 2 mM L-glutamine (Sigma), 25 mg/ml transferrin (Sigma), 100 mg/ml streptomycin and 100 U/ml penicillin (Sigma). On day 14, CBZ- bulks were separated into CD4 and CD8 cells by anti-human CD8 magnetic beads (Miltenyi Biotec) via positive selection, and isolated as monoclonal populations through limiting dilution in 96-well. TCCs were expanded in 96-well plates via repetitive mitogen driven expansion (PHA) in the presence of irradiated allogeneic PBMCs from healthy donors every 14 days, punctuated by maintenance feeding with IL-2 supplemented R9 medium every 2 days. Well-growing clones were picked from the serial dilution, further expanded across 4 wells for a further 14 days.

For assessing drug responsiveness clones exhibiting adequate expansion were drawn and split into 4 wells in an additional testing plate (5×10^4 cells per well). TCCs were incubated with 1×10^4 irradiated APC per well in R9 medium with and without the drug in duplicate in 96-well plate. The plate was incubated at 37°C, 5% CO_2 for 48 hours. 0.5 μCi [^{3}H]-thymidine was then added to each well, and incubated for an additional 16 hours. Proliferation activity was assessed through incorporated tritiated thymidine measured by a Beta scintillation counter (Perkin Elmer) as a function of counts per min (cpm). CBZ-specific proliferative response was defined as a stimulation index (SI; equal to cpm with drug divided by cpm without drug) of equal or greater than 1.5. SI $\geq$ 2 and $\geq$ 4 was interpreted as good and strong response respectively.

2 Cellular characterization of drug-specific T-cell clones

CD4/CD8 phenotype of drug-specific T-cell clones was tested using anti-CD4-FITC antibody (Miltenyi Biotec) and anti-CD8-antibody (Miltenyi Biotec) according to manufacturer instructions. A BD FACS Canto II flow cytometer (BD biosciences) was used for the fluorescence analysis. For cytokine release assays, the IFN-γ ELISpot (Abcam) analysis was done in duplicate by incubating T-cell clones (5×10^4) with irradiated autologous antigen-presenting cells (EBV-transformed B-cells 1×10^4) for 48 hours (37°C, 5% CO_2) before read-out analysis, conducted according to manufacturer's instructions.

3 HLA restriction assays

The HLA restriction of drug-specific T-cell clones was determined through HLA mismatching and HLA blocking analyses. HLA mismatching analysis was conducted using 10-15 irradiated allogenic EBV-transformed B-cell lines as APCs. The panel was selected and evaluated based on the expression of candidate HLA alleles matching the drug-specific T-cell response.

The HLA blocking experiments utilized antibodies against HLA class I (W6/32, Abcam), HLA class II (6C6, Abcam), and a panel of locus-specific antibodies against HLA-DP (B7/21), HLA-DQ (SPV-L3), and HLA-DR (L243, Abcam). We pre-treated the APCs and TCCs with 3 μl of locus-specific

antibody against HLA-DP, HLA-DQ, and HLA-DR (Abcam) for 1 hour before addition of drug or media control to evaluate proliferation. Isotype-matched control antibodies for HLA class I and class II (Abcam) were established alongside these assays as internal controls.

4 RNA isolation for TCR sequencing

TCR from mRNA of the drug-specific T-cells were sequenced to explore the characteristics of drug-specific TCRs. To promote cellular proliferation and expression, the clones were thawed and restimulated using irradiated random donor PBMCs and fed with IL-2-supplemented R9 for 5-7 days before RNA isolation. Only T-cell clones with a cell count $\geq 5\times10^6$ and ≥50% viability was chosen for subsequent RNA isolation.

RNA isolation was performed using the RNeasy Mini Kit as per manufacturer instruction (Qiagen). Briefly, T-cell clones were washed with PBS and lysed by vortexing in ethanol. The lysate was then transferred to a spin column, washed twice, treated with DNase, washed again twice, and then eluted in nuclease-free water. All RNA samples were checked for their quality and quantity using a nanodrop (Thermo Scientific) and Fragment Analyzer (Agilent). Only RNA with an A260/A280 ratio of equal to or more than 1.7, total RNA greater than 700ng, RNA quality number (RQN) value ≥ 9, and showing no signs of degradation, was chosen for further TCR library preparation and SMRT sequencing. An overall workflow for the TCR sequencing experiment is shown in Supplementary Figure S1.

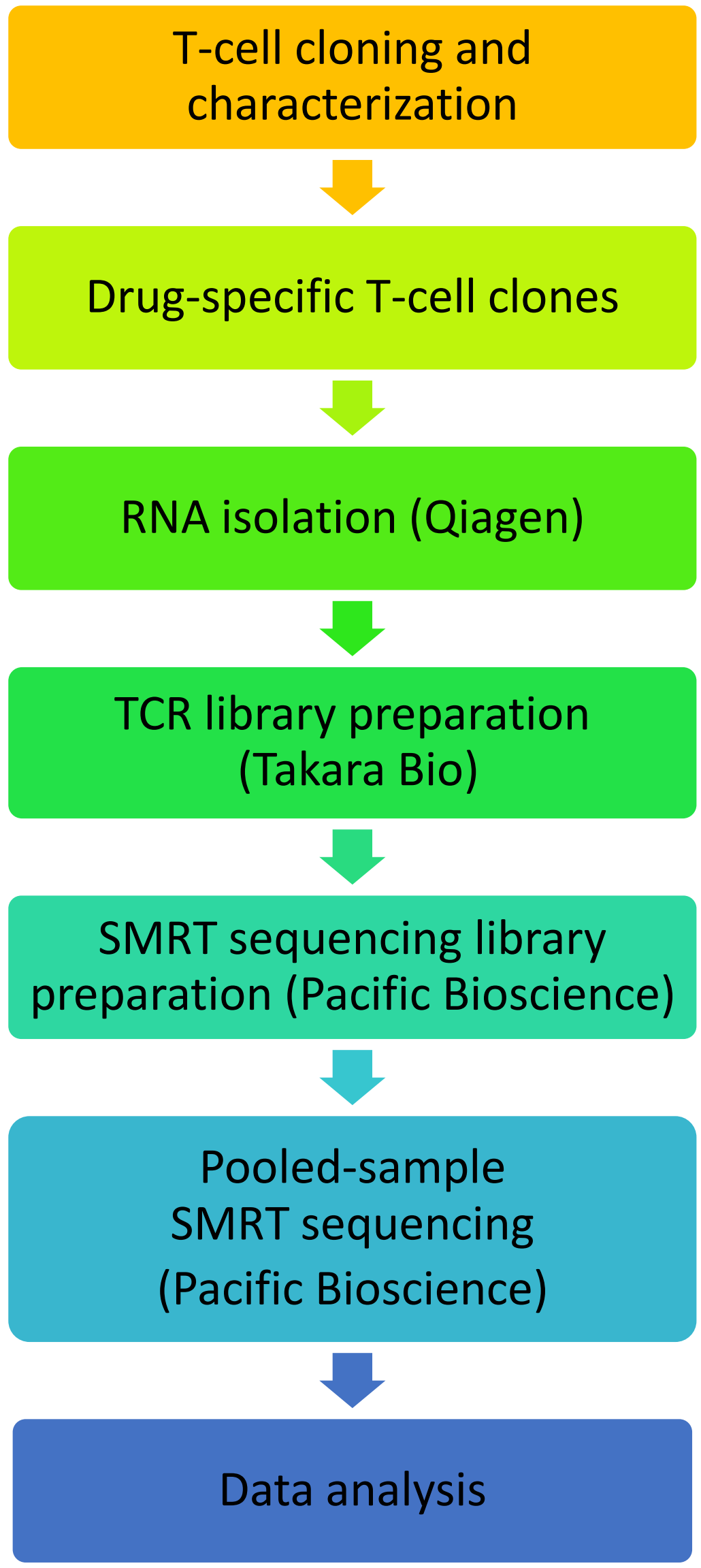

Supplementary Figure S1 Overall workflow of TCR sequencing experiment. The TCR library preparation, SMRT sequencing library preparation, and pooled SMRT sequencing were done collaboratively with the Centre for Genomic Research. SMRT: Single Molecule, Real-Time sequencing.

5 Library preparation of TCR transcript

After quality control analyses, all isolated RNA samples were used for TCR sequencing library preparation by SMARTer Human TCR a/b Profiling Kit (Takara Bio) following manufacturer's instruction. This method consisted of an initial reverse transcription reaction and subsequent 2-step semi-nested PCR.

In the reverse transcription reaction, the SMART technology (Switching Mechanism at 5' End of RNA template) was applied to add non-templated SMART-Seq v4 Oligonucleotide to the 5' end of the mRNA template and capture the V(D)J region of the TCR transcripts. The isolated RNA was used as a template of a reverse poly-deoxythimidine (dT) primer and the SMART-Seq v4 oligonucleotide was added to the 5' end of the RNA template. A cDNA with an additional oligonucleotide sequence was generated from template RNA in this step.

Subsequently, in a semi-nested PCR, the cDNA was amplified in the first PCR by a forward primer that binds to the sequence of added oligonucleotide and a reverse primer that binds the C region of the TCR. This reaction amplified the whole V region and a part of the C region of the TCR transcript. In the second PCR, the amplicon was amplified using a pair of a forward and reverse primers with unique index sequences that bind to the 5' and 3' ends of the amplicon. As a result, a TCR library with an index barcode on both ends was ready after this step (Supplementary Figure S2).

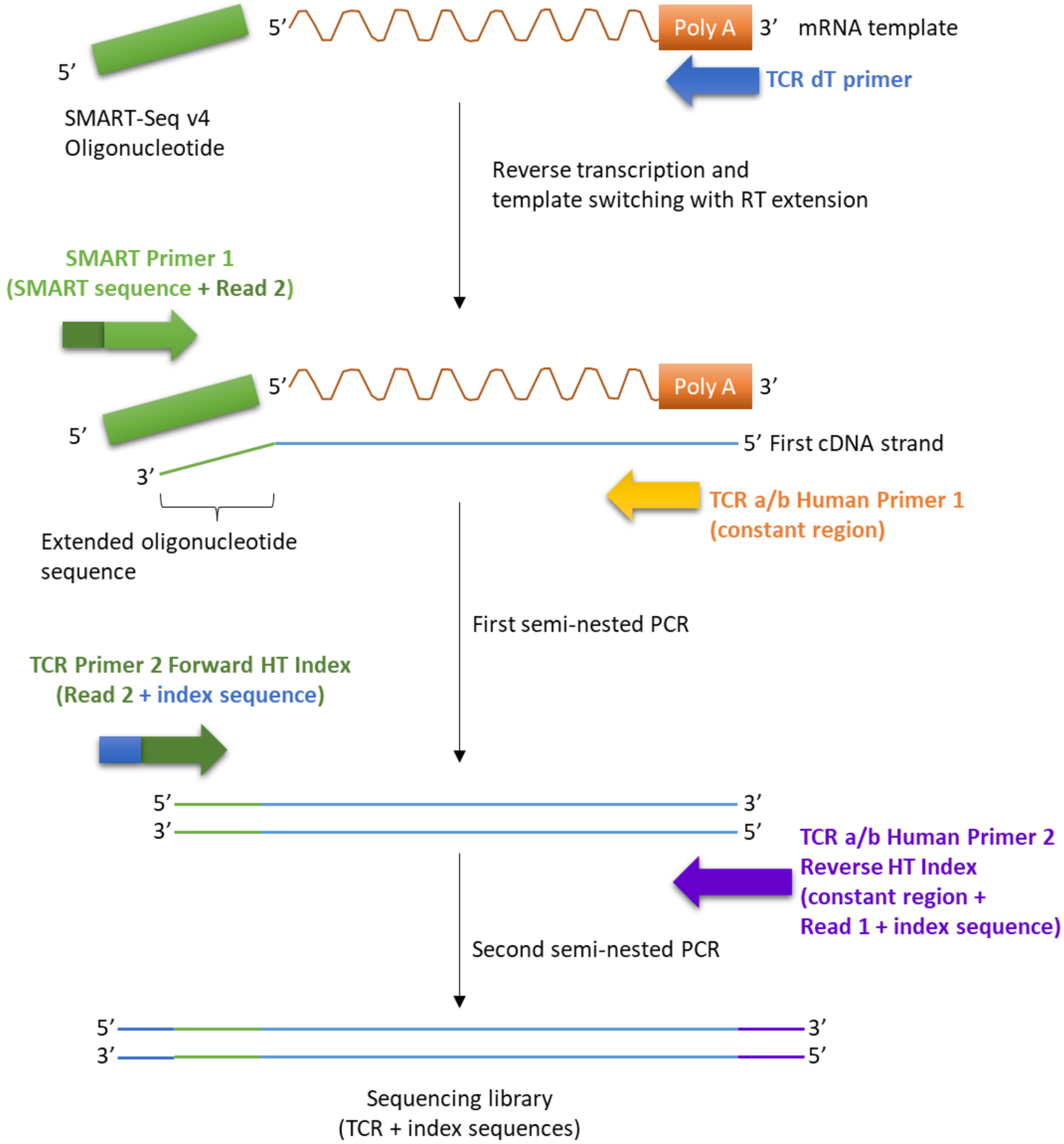

Supplementary Figure S2 TCR library preparation by SMARTer Human TCR a/b Profiling kit (Takara Bio). Adapted from the SMARTer® Human TCR a/b Profiling Kit User Manual (2016). The prepared library covers both variable region and constant region, and also contains index sequences on both the 5' and 3' ends. cDNA: complementary DNA, dT: deoxythymidine, RT: reverse transcription, HT: high throughput.

6 Long-read sequencing of the TCR libraries

For long-read sequencing of the TCR library, the Single Molecule, Real-Time (SMRT®) sequencing method (Pacific Biosciences) was used in this study. The SMRT sequencing library was prepared by treating the sequencing template with nuclease and ligated with barcoded SMRT bell adaptors. After ligation, the template obtained a topologically circular structure which allows continuous long-read sequencing on both sense and antisense strands. In this experiment, the SMRT library of each sample was prepared separately, and then pooled and processed together in a single long-read sequencing run.

In the sequencing reaction, every single molecule of the sequencing library was immobilized into a small well called "zero-mode waveguide" and was used as a template of the polymerase sequencing reaction. The polymerase enzyme incorporated labelled nucleotides resulted in light emission, and generated a long repetitive sequencing read from the template. The raw data was then processed into a consensus read called Circular Consensus Sequence (CCS). The obtained pooled sequencing data was subsequently demultiplexed in the data analysis step. The overall process of SMRT sequencing is shown in Supplementary Figure S3.

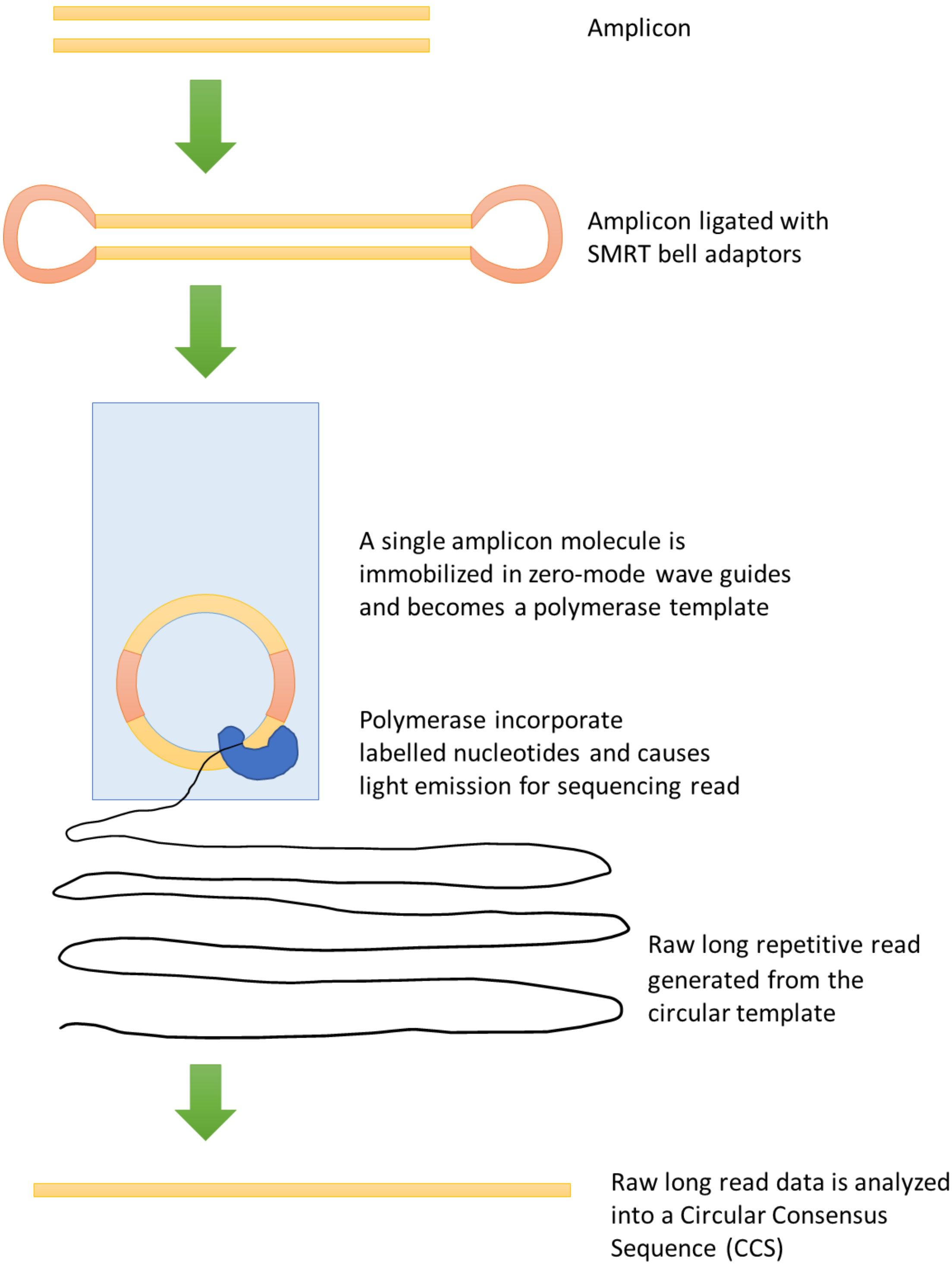

Supplementary Figure S3 SMRT long-read sequencing work pipeline.

7 Bioinformatic analysis

The raw sequencing data was processed using a bioinformatic pipeline (Figure 4.4). The raw data was demultiplexed based on SMRT barcodes. The demultiplexed data for each clone was quality-checked before subsequent analyses. Then, the TCR characteristics (including VDJ usage, CDR3 characteristics, clonotype frequency) were identified using MiXCR. Cluster analysis and phylogenetic tree analysis was done using VDJtools. Alignment and comparison of the CDR3 characteristics were done using WebLogo, T-Coffee, and Jalview.

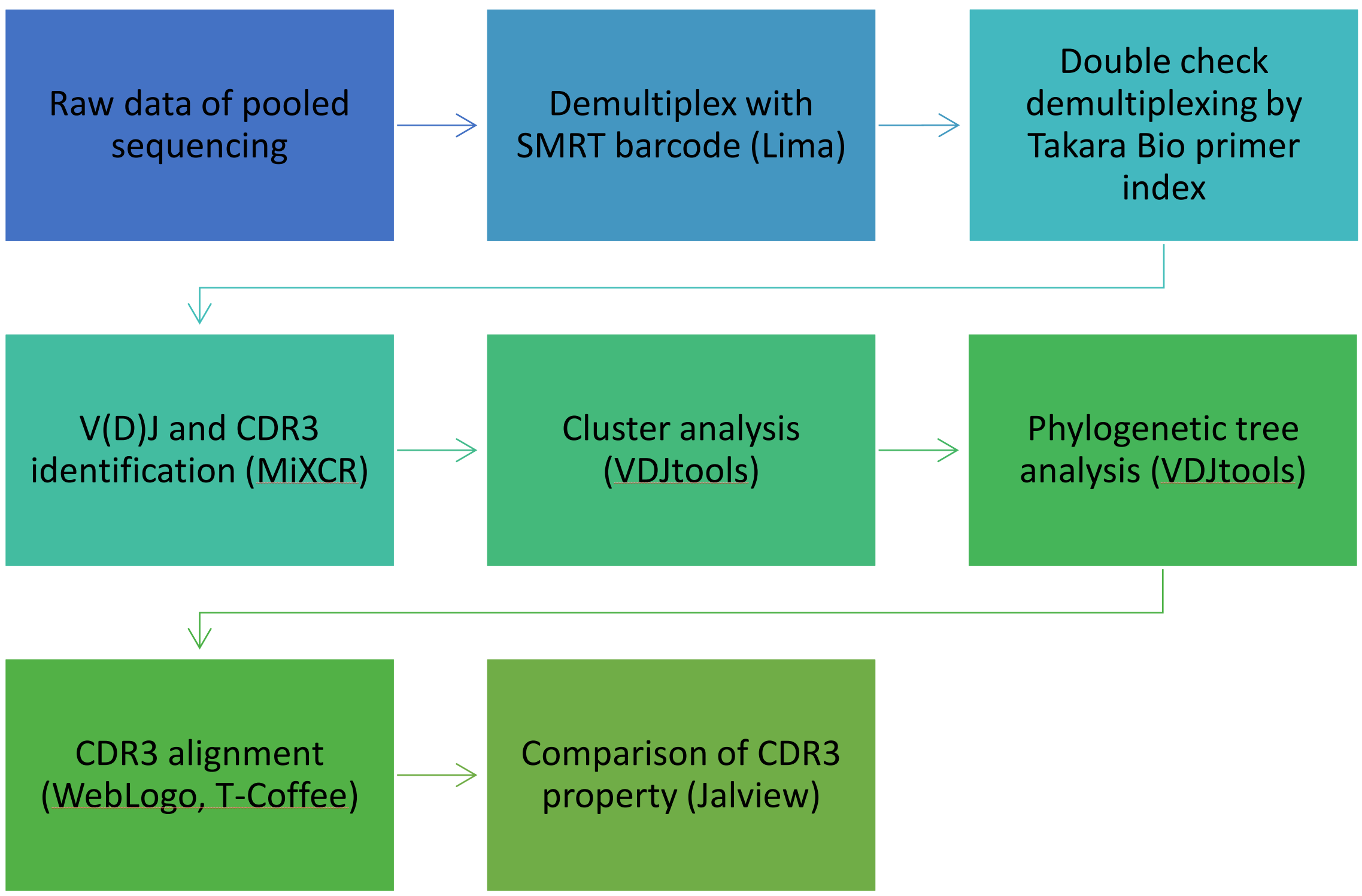

Supplementary Figure S4 Data analysis workflow

7.1 Demultiplexing of the pooled sequencing data

In the demultiplexing process, the pooled sequencing data were extracted based on the SMRT primer index and sequences of each sample split into a separate file. The Lima package (the PacBio Barcode Demultiplexer and Primer Remover) was used for this process (available at https://lima.how/). The demultiplexed data were quality checked by checking sequencing reads and examining the presence of Takara Bio primer barcodes in demultiplexed data.

7.2 V(D)J segment and CDR3 identification

The VDJ gene usage and CDR3 sequence of each clone was identified from the demultiplexed data by using the MiXCR tool (1, 2). In this analysis, the demultiplexed sequences were aligned to reference TCR genes (including V, D, J, and C gene segments), assembled into a clonotype, and identified CDR3 sequences of each read. Sequencing reads with similar TCR characteristics were assembled into a clonotype. The aligned sequencing reads were reported in this step. The "analyze" command was used for executing all of this process systematically. The processed TCR data were exported for further downstream analyses.

7.3 Cluster analysis and phylogenetic tree analysis

The exported aligned TCR data were used for cluster analysis and phylogenetic tree analysis by the VDJtools software (3). The "CalcPairwiseDistance" and "ClusterSamples" commands were used to evaluate clonotype similarities between samples. The result was illustrated as a multi-dimensional scaling plot and hierarchical clustering plot which represents relatedness and pairwise distances between samples.

7.4 CDR3 alignment

For high-resolution CDR3 analyses, the CDR3 residue data of each clone were extracted and all non-productive TCR transcripts were excluded before further CDR3 analyses. All CDR3s were grouped and aligned based on their drug-responsiveness properties. The CDR3s with a similar length were compared using WebLogo (4).

The CDR3 with different lengths were aligned by the T-Coffee tool on the EMBL-EBI database (5). For CBZ-specific T-cell clones, the CDR3 characteristics were classified and compared based with the CBZE cross-reactivity and HLA restriction properties. For the SMX-specific clones and DDS-specific clones, as the sample size was limited, the CDR3 characteristics were compared regardless of cross-reactivity and HLA restriction properties.

7.5 Comparison of physicochemical properties of the CDR3s

After the CDR3 alignment, the physicochemical property of CDR3 residues was compared using Jalview software (6). The conservation of each residue on the alignment was also quantitatively calculated by the AMAS method (7). The conservation score ranges from 0 to 11 based on the conserved property of a residue. The lowest score of 0 represents no conserved residue while the highest score of 11, indicated by an asterisk (*) in the alignment, represents a fully conserved amino acid residue. A score of 10, indicated by a + in the alignment, represents a residue with mutations that conserved all properties.

In the comparison of physicochemical properties of CDR3s, the alignment was coloured based on the Clustal X colouring scheme to highlight conserved residues and depict differences in properties among the TCRs (8). The colouring criteria of Clustal X scheme are shown in Supplementary Table S1.

Supplementary Table S1 Clustal X colouring scheme. This table was adapted from Jalview documentation at www.jalview.org/help/html/colourSchemes/clustal.html.

**Category**	**Colour**	**Residue**	**%Threshold of residue group at the position**
Hydrophobic	Blue	A, I, L, M, F, W, V, C	>60% are WLVIMAFCHP combined
Positive charge	Red	K, R	>60% are KR combined, or<br>>80% are K or R or Q individually
Negative charge	Magenta	E	>60% are KR combined, or<br>>50% are QE combined, or<br>>85% are E or Q or D individually
		D	>60% are KR combined, or<br>>85% are E or Q or D individually, or<br>>50% are ED combined
Polar	Green	N	>50% are N, or<br>>85% are N or Y individually
		Q	>60% are KR combined, or<br>>50% are QE combined, or<br>>85% are Q or E or K or R individually

		S, T	>60% are WLVIMAFCHP combined, or >50% are TS combined, or >85% are S or T individually
Cysteines	Pink	C	>85% are C
Glycines	Orange	G	>0% are G
Prolines	Yellow	P	>0% are P
Aromatic	Cyan	H, Y	>60% are WLVIMAFCHP combined, or >85% are W or Y or A or C or P or Q or F or H or I or L or M or V
Unconserved	White	any, gap	If none of the above criteria are met

8 Supplementary references

1. Bolotin DA, Poslavsky S, Mitrophanov I, Shugay M, Mamedov IZ, Putintseva EV, Chudakov DM. MiXCR: software for comprehensive adaptive immunity profiling. Nat Methods. 2015;12(5):380-1.

2. Bolotin DA, Poslavsky S, Davydov AN, Frenkel FE, Fanchi L, Zolotareva OI, et al. Antigen receptor repertoire profiling from RNA-seq data. Nat Biotechnol. 2017;35(10):908-11.

3. Shugay M, Bagaev DV, Turchaninova MA, Bolotin DA, Britanova OV, Putintseva EV, et al. VDJtools: Unifying Post-analysis of T Cell Receptor Repertoires. PLoS Comput Biol. 2015;11(11):e1004503.

4. Crooks GE, Hon G, Chandonia JM, Brenner SE. WebLogo: a sequence logo generator. Genome Res. 2004;14(6):1188-90.

5. Madeira F, Park YM, Lee J, Buso N, Gur T, Madhusoodanan N, et al. The EMBL-EBI search and sequence analysis tools APIs in 2019. Nucleic Acids Res. 2019;47(W1):W636-w41.

6. Waterhouse AM, Procter JB, Martin DM, Clamp M, Barton GJ. Jalview Version 2--a multiple sequence alignment editor and analysis workbench. Bioinformatics. 2009;25(9):1189-91.

7. Livingstone CD, Barton GJ. Protein sequence alignments: a strategy for the hierarchical analysis of residue conservation. Comput Appl Biosci. 1993;9(6):745-56.

8. Thompson JD, Gibson TJ, Plewniak F, Jeanmougin F, Higgins DG. The CLUSTAL_X windows interface: flexible strategies for multiple sequence alignment aided by quality analysis tools. Nucleic Acids Res. 1997;25(24):4876-82.
